# Expanding our knowledge of Synurales (Chrysophyceae) in Florida, United States: Proposing three novel *Mallomonas* taxa

**DOI:** 10.1111/jpy.70062

**Published:** 2025-07-29

**Authors:** Petr Knotek, Martin Pusztai, Iva Jadrná, Pavel Škaloud

**Affiliations:** ^1^ Faculty of Science, Department of Botany Charles University Prague Czech Republic; ^2^ Institute for Nanomaterials, Advanced Technologies and Innovation Technical University of Liberec Liberec Czech Republic; ^3^ Faculty of Science, Humanities and Education Technical University of Liberec Liberec Czech Republic

**Keywords:** adaptation, biodiversity hotspot, electron microscopy, Florida, new species, phylogeny, siliceous scales, Synurales, taxonomy

## Abstract

We conducted a survey of Synurales diversity in Florida (United States), focusing on two established hotspots—Ocala National Forest and Myakka River State Park—and two previously unexplored sites—Manatee Lake and Lake Lee. Using transmission electron microscopy (TEM), we identified 69 species, increasing the total number recorded from Florida to 90. Among these, three species—*Mallomonas cornea* sp. nov., *M. laureana* sp. nov., and *M. joergenii* sp. nov.—have been newly described based on detailed morphological and molecular analyses. We have also proposed a new combination, *M. poseidonii* comb. et stat. nov., and established a new section, Corneae sect. nov., based on molecular phylogenetics. Our findings revealed an independent emergence of ribbed scale shield ornamentation in *M. joergenii* sp. nov. and suggested that internal scale reticulation in *M. laureana* sp. nov. may be an adaptation to high UV irradiance in tropical environments. This study underscores the value of DNA sequence data in resolving taxonomic ambiguities, elucidating evolutionary patterns, and enhancing species recovery from type localities.

**Abbreviations**
MCMC: Markov chain Monte Carlo
ML: maximum likelihood
nu: nuclear
pt: plastid
SDSF: standard deviation of split frequencies
SEM: scanning electron microscopy
TEM: transmission electron microscopy

## INTRODUCTION

Protists, a diverse group of eukaryotic microorganisms, play crucial roles in various ecological processes, including nutrient cycling and primary production, and are a food source in aquatic and terrestrial ecosystems (Adl et al., 2019). Despite their ecological significance, the diversity and biogeography of protists remain considerably understudied (Pinseel et al., 2024). This knowledge gap hinders our understanding of ecosystem dynamics and the full extent of biodiversity. Advances in molecular systematics using DNA sequence data have begun to reveal the vast genetic diversity among protists, suggesting that many species have yet to be discovered (Caron & Countway, 2009). Furthermore, understanding their distribution patterns is essential for predicting ecosystem responses to environmental change and for conservation efforts (Cavalier‐Smith, 2004). By intensifying research on protist diversity and their biogeography, scientists can gain deeper insights into ecological interactions, evolutionary processes, and ecosystem resilience in the face of global change (Weisse, 2017).

It appears that species diversity is understudied, even in protist groups, where species‐specific morphologies allowed a large number of species to be described well before the development of DNA sequencing techniques (Škaloud et al., 2013). Among them is the order Synurales, which contains the flagellates of the genera *Mallomonas*, *Synura*, and *Neotessella*. These species form characteristic shells consisting of siliceous scales and varying numbers of bristles on their cell surfaces (Andersen, 1987). The continuous rise in newly described species is a good indicator of our incomplete understanding of the diversity of these flagellates (Hao et al., 2024). This is true even though DNA‐based molecular characterization is still used less frequently to describe new species within this group than conventional morphological techniques (Gusev, Ignatenko, & Yatsenko‐Stepanova, 2024; Piątek & Łukaszek, 2016; Safronova et al., 2024). It is not surprising that many of the recently described new species originated from undersampled and previously difficult‐to‐reach locations, primarily in the tropics and subtropics (Gusev, Martynenko, et al., 2023; Kapustin et al., 2019; Piątek, 2015).

In the United States, the many accessible aquatic habitats in Florida are important for studying the diversity and biogeography of algae, including scaled chrysophytes (Whelden, 1941; Pollman & Canfield Jr., 1991; Siver & Lott, 2006). The first record of the occurrence of organisms of the order Synurales in Florida was in Wujek, 1983, and the first article directly addressing the diversity of this group was published by Wujek just 1 year later (Wujek, 1984). This initial study began a series of 11 further publications showing how species‐rich Florida is regarding this group of organisms (Siver, 1994, 1999, 2002a, 2002b; Siver & Lott, 2006; Siver & Wujek, 1993, 1999; Wujek & Bland, 1988, 1991; Wujek & Moghadan, 2001; Wujek & Siver, 1997). To date, 22 Florida counties have been sampled, with a total of 66 species and subspecific taxa observed and nine new taxa described. Most of those taxa have been described from Ocala National Park (*Mallomonas binocularis, M. caerula, M. corymbosa* var. *poseidonii, M. delanciana, M. ocalensis, M. transsylvanica* f. *curvata*, and *M. wujekii*), with the remaining two from Myakka River State Park (*M. matvienkoae* var. *myakkana*) and Hickey Creek Mitigation Park (*M. parvula* var. *nichollsii*; Siver, 1991, 1994, 1999, 2002a, 2002b; Siver & Lott, 2006; Wujek & Bland, 1988). The uniqueness of this area has also been demonstrated by the fact that three of these new taxa have so far been recorded only in Florida (*M. matvienkoae* var. *myakkana, M. parula* var. *nichollsii*, and *M. transsylvanica* f. *curvata*), and four have been observed outside Florida only in the states of North and South Carolina (*M. caerula, M. delanciana, M. ocalensis*, and *M. wujekii*; Dillard, 2007; Wujek et al., 2004). Furthermore, it can be assumed that a significant amount of the diversity is still unknown due to the potential rarity of certain species. This is evident from the fact that 13 taxa, including some that are widely distributed, have been observed just once in Florida (*M. annulata, M. corcontica, M. heterospina, M. leboimei, M. muskokana, M. pumilio, M. punctifera, M. sphagniphila, M. transsylvanica* f. *curvata, Synura mammillosa, S. mollispina, S. praefracta*; Dillard, 2007).

The main goal of this study was to continue long‐term research on the diversity of the order Synurales in Florida and to find out if: (i) the overall diversity had changed (i.e., were there any taxa that were either recorded for the first time or could not be recorded again); (ii) new species would be discovered (i.e., even after a great deal of research, diversity is not well documented); (iii) the taxa described from Florida would be observed again and, if so, whether they occurred in the vicinity of their type localities. For this purpose, a total of 13 samples were collected, and their overall diversity was determined. Eleven sites, three in Myakka River State Park and eight in Ocala National Forest, were sampled to track the presence of eight previously described taxa in these areas. Samples were also collected from the remaining two sites, Lake Manatee and Lake Lee, with the additional objective of determining whether they contained species not yet reported in Florida. In the case of species not yet known to science, efforts were made to isolate them into culture to obtain molecular data for a proper description.

## MATERIALS AND METHODS

### Collection, isolation, and cultivation of strains

Phytoplankton samples were collected from 13 waterbodies in Florida between October 10 and 12, 2022 (Table 1). Samples were collected using a plankton net with a mesh size of 20 μm. The standard measurements of water temperature, pH, and specific conductivity were obtained using a combined pH/conductometer (WTW 340i; WTW GmbH, Weilheim, Germany). Two milliliters of the sedimented samples were fixed with formaldehyde, and a portion of the remaining volume was examined on the same day of sampling with an Olympus CX 31 light microscope. The individual *Mallomonas* flagellates were isolated by micropipetting. Each cell was placed in a separate well of a 96‐well polypropylene plate filled with approximately 300 μL of modified liquid WC medium. The medium contained twice the concentration of Na_2_SiO_3_•9H_2_O (56.8 mg · L^−1^) and was buffered with either MES (for strains US58L and US58AB and strain US53J; pH ≈ 6.7) or TES (for strain US46H and strains US55E and US55K; pH ≈ 7.5). In the laboratory, well‐grown cultures were transferred from the wells to 50‐mL Erlenmeyer flasks filled with the same medium. The cultures were maintained at a constant temperature of 24°C and under constant illumination of 50 μmol photons · m^−2^ · s^−1^ (TLD 18 W/33 fluorescent lamps, Philips, Amsterdam, the Netherlands). Light intensity was measured with a Wallz ULM‐500 radiometer equipped with a Spherical Micro Quantum Sensor.

**TABLE 1:** Physical and chemical data for 13 Florida study sites.

Name	GPS coordinates	Temperature [°C]	pH	SC [μS · cm^−1^]	Sampling date
Lake Dorr	29°00′47.6″ N, 81°38′05.9″ W	29.2	5.9	62	10.10.2022
Near Echo Lake	29°06′23.2″ N, 81°38′53.3″ W	26.6	5.6	37	10.10.2022
Echo Lake	29°06′15.6″ N, 81°39′02.4″ W	26	4.4	32	10.10.2022
Grasshopper Lake	29°08′04.0″ N, 81°37′11.3″ W	30.6	4	40	10.10.2022
Cowpen Pond	29°01′18.0″ N, 81°27′32.2″ W	26.6	4.7	35	11.10.2022
Blue Sink	29°03′39.6″ N, 81°40′12.5″ W	26.9	5.3	27	11.10.2022
Laura Lake	29°25′02.3″ N, 81°40′54.2″ W	29.1	4.8	111	11.10.2022
Penner Lake	29°29′27.0″ N, 81°49′18.8″ W	29	5.1	42	11.10.2022
Oxbow Lake	27°14′43.9″ N, 82°18′28.7″ W	29.1	6	135	12.10.2022
Myakka River	27°14′44.1″ N, 82°18′24.6″ W	27.3	6	122	12.10.2022
Canal near Myakka River	27°14′14.8″ N, 82°18′53.7″ W	27.4	6	136	12.10.2022
Lake Matetee	27°28′53.6″ N, 82°20′26.7″ W	26.6	5.5	78	12.10.2022
Lake Lee	27°58′40.6″ N, 81°36′30.3″ W	27.9	5.2	169	12.10.2022

### Morphological investigations

Observation and photography of live *Mallomonas* cells were performed using an Olympus BX51 light microscope equipped with Nomarski interference contrast and a Canon EOS 7 camera. For observation of the siliceous scales and bristles by transmission electron microscopy (TEM), samples from each culture were prepared by drying on Formvar‐coated copper grids (SPI Supplies 3220C, West Chester, United States). Fixed samples from the localities were left in 90°C hydrogen peroxide for 15 min and then washed four times with deionized water and centrifuged before plating on the grids. The grids were rinsed by repeated transfer of the grid in four drops of deionized water placed on the hydrophobic surface of a Parafilm strip (PM‐996, Bemis, United States). The dried grids were examined using a JEOL 1011 transmission electron microscope (JEOL USA, Inc., Peabody, United States) equipped with a Veleta CCD camera and acquisition software (Olympus Soft Imaging Solution GmbH, Münster, Germany). For scanning electron microscopy (SEM), selected cultures were washed by repeated centrifugation in deionized water, air‐dried on coverslips, attached to aluminum stubs with double‐sided adhesive carbon tape, and coated with platinum and palladium (4:1) for 95 seconds. The samples were coated with a thin layer of gold–palladium using a Bal‐Tec SCD 050 sputter coater (Bal‐Tec, Balzers, Liechtenstein) and then observed using a JEOL JSM‐IT800 field emission scanning electron microscope (JEOL Ltd., Tokyo, Japan). Measurements were performed using ImageJ version Fiji (Schindelin et al., 2012) on a minimum of 20 cells per strain.

### Sequencing and phylogenetic analysis

For DNA isolation, approximately 90 mL of well‐grown cultures was harvested by centrifugation and frozen at −80°C overnight. Then, the genomic DNA was isolated using the DNeasy Blood & Tissue Kit (Qiagen, Venlo, The Netherlands). DNA libraries were prepared using the xGen™ DNA Library Prep EZ UNI Kit and sequenced on the Illumina NextSeq platform with 150 bp paired‐end reads. Sequenced reads were trimmed with Trimmomatic v. 0.32, with the Phred quality scores of 33. De novo assembly of plastid genomes was performed in GetOrganelle v. 1.7.7.0, using *Mallomonas splendens* (NC_040135) and *Synura uvella* (NC_040134) chloroplast genome sequences as a seed. The genomes were assembled using default settings, with different k‐mer sizes (21, 45, 65, 85, and 105). Gene prediction was carried out in MFannot (http://megasun.bch.umontreal.ca/apps/mfannot/), and the plastid LSU rDNA, *psa*A, and *rbc*L genes were subtracted for the phylogenetic analysis. Nuclear SSU and LSU rDNA genes were extracted from the trimmed sequenced reads by BWA v. 0.7.3a (Li & Durbin, 2009) using a local alignment of rDNA sequences. The loci of strain US46H were obtained by Sanger sequencing, following the methodology described in Škaloud et al. (2020).

Newly obtained sequences were supplemented by several sequences deposited in the GenBank database (Table S1). The sequences were aligned using MAFFT version 7 under the Q‐INS‐I strategy (Katoh & Standley, 2013). DNA alignments are freely available on Mendeley Data: https://doi.org/10.17632/cb73kzfrww.1. The loci of nuclear (nu) SSU rDNA, nu LSU rDNA, plastid (pt) LSU rDNA, pt. *rbc*L, and pt. *psa*A genes were concatenated, yielding a robust alignment of 9941 bases, 62 *Mallomonas* strains, and four outgroup taxa. Prior to performing the concatenated phylogenetic analysis, maximum likelihood (ML) analyses were performed separately for each locus in RAxML 8.1.20 (Stamatakis, 2014) to verify there were no obvious topological incongruencies among the loci. The phylogenetic tree was inferred by Bayesian inference in MrBayes v3.2.6 (Ronquist et al., 2012) on a concatenated dataset divided into nine partitions (ribosomal genes, and codon‐partitioned protein‐encoded genes), using the GTR + Γ + I evolutionary model. Two parallel Markov chain Monte Carlo (MCMC) runs, with one cold and three heated chains, were run for 40 million generations. Trees and parameters were sampled every 100 generations. The convergence of the two cold chains was assessed during the run by calculating the average standard deviation of split frequencies (SDSF). The first 25% of the trees were discarded as burn‐in in each run. The bootstrap analysis was performed by ML in RAxML 8.1.20, using two independent runs, 1000 pseudoreplicates, and the default GTR + Γ evolutionary model.

## RESULTS

### Species diversity

**FIGURE 1:**
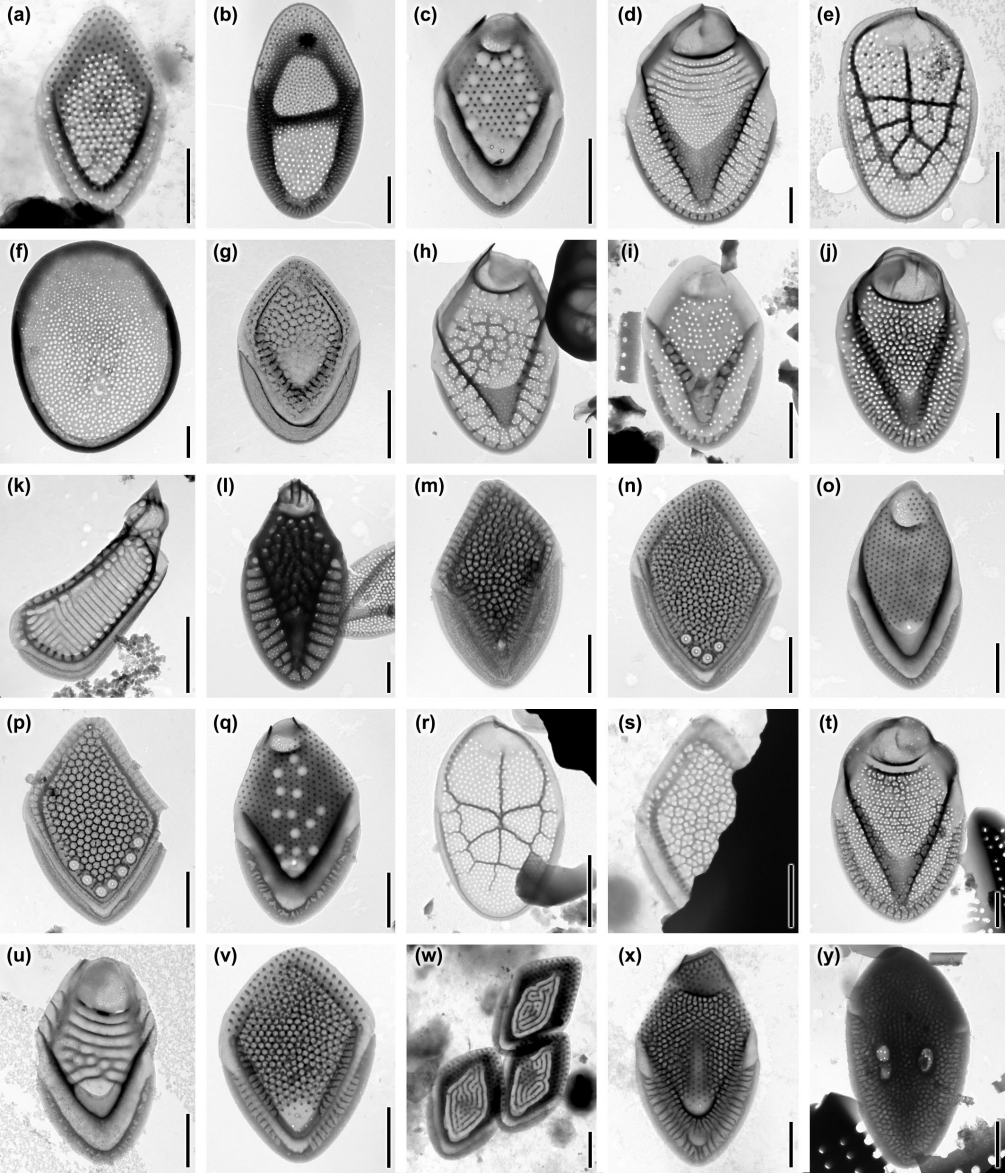
*Mallomonas* taxa identified in natural populations. (a) *M. annulata*, (b) *M. bangladeshica*, (c) *M. binocularis*, (d) *M. caerula*, (e) *M. canina*, (f) *M. caudata*, (g) *M. cornea* sp. nov., (h) *M. crassisquama*, (i) *M. cristata*, (j) *M. cyathellata*, (k) *M. dickieae*, (l) *M. duerrschmidtiae*, (m) *M. favosa*, (n) *M. foveata*, (o) *M. furtiva*, (p) *M. gemina*, (q) *M. guttata*, (r) *M. hindonae*, (s) *M. hualvensis*, (t) *M. intermedia*, (u) *M. joergenii* sp. nov., (v) *M. kornevae*, (w) *M. labyrinthina*, (x) *M. laureana* sp. nov, (y) *M. lychenensis*. Scalebar = 1 μm.

**FIGURE 2:**
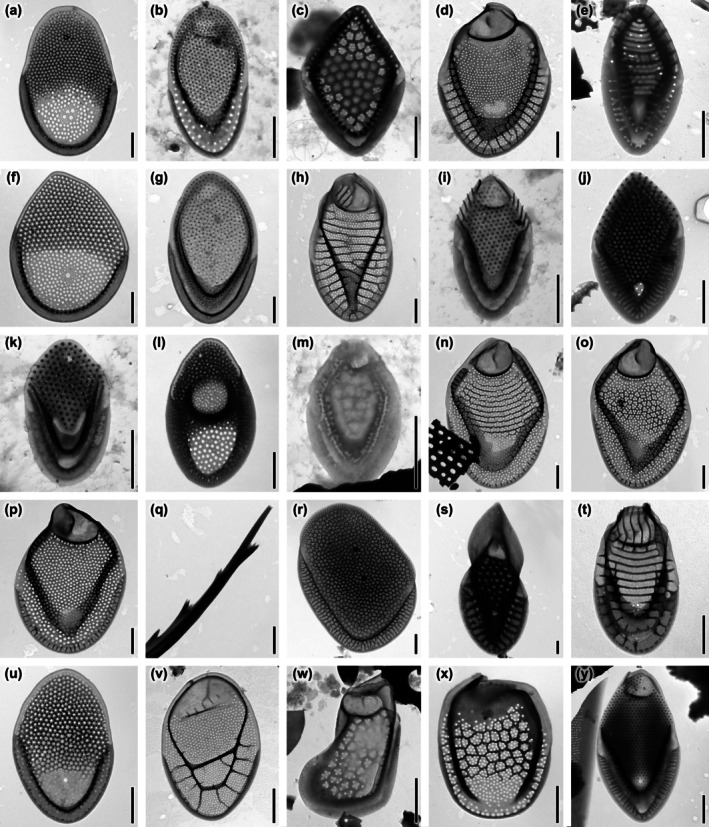
*Mallomonas* taxa identified in natural populations. (a) *M. matvienkoae* var. *myakkana*, (b) *M. multisetigera*, (c) *M. munda*, (d) *M. muskokana*, (e) *M. ocalensis*, (f) *M. okhapkinii*, (g) *M. ouradion*, (h) *M. paludosa*, (i) *M. papillosa*, (j) *M. parana*, (k) *M. parvula*, (l) *M. peronoides*, (m) *M. pillula* var. *valdiviana*, (n) *M. portae‐ferreae*, (o) *M. portae‐ferreae* var. *reticulata*, (p) *M. poseidonii* comb. et stat. nov. – scale, (q) *M. poseidonii* comb. et stat. nov.—bristle, (r) *M. pseudobronchartiana*, (s) *M. pseudocoronata*, (t) *M. pseudocratis*, (u) *M. pseudomatvienkoae*, (v) *M. pugio*, (w) *M. pumilio*, (x) *M. punctifera* var. *brasiliensis*, (y) *M*. cf. *rasilis*. Scalebar = 1 μm.

**FIGURE 3:**
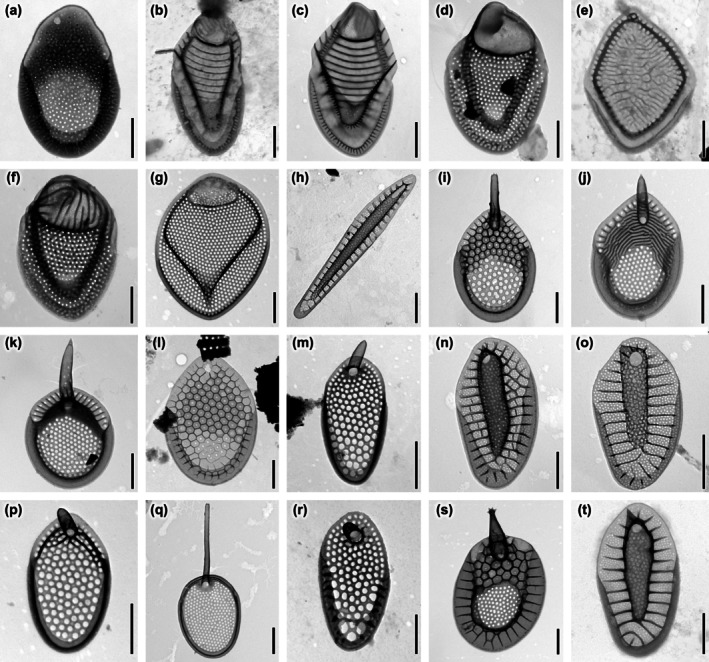
*Mallomonas* (a‐g) and *Synura* (h‐t) taxa identified in natural populations. (a) *M. stellata*, (b) *M. striata*, (c) *M. striata* var. *serrata*, (d) *M. tonsurata*, (e) *M. torquata*, (f) *M. wujekii*, (g) *Mallomonas* sp., (h) *S. americana*, (i) *S. curtispina*, (j) *S. echinulata*, (k) *S. leptorrhabda*, (l) *S. mollispina*, (m) *S. papillosa*, (n) *S. petersenii*, (o) *S. praefracta*, (p) *S. prowsei*, (q) *S. sphagnicola*, (r) *S. synuroidea*, (s) *S. uvella*, (t) *S. vinlandica*. Scalebar = 1 μm.

A total of 69 taxa belonging to Synurales were observed at the 13 sampled sites in Florida (Table S2). Out of them, 56 belonged to the genus *Mallomonas*; the remaining 13 were classified under the genus *Synura* (Figures 1, 2, 3). The number of taxa in each site ranged from seven to 32, with a mean of 15. In the study, 21 taxa previously known from Florida were not observed, but 16 previously unknown taxa were recorded from this area. Additionally, three new species were identified and are herein described. Considering this, there are now 90 taxa of Synurales that have been observed in the state of Florida. In addition, an undescribed *Mallomonas* taxon was also observed (Figure 3g). Nine organisms, *M. furtiva, M. guttata, M. okhapkinii, S. curtispina, S. echinulata, S. leptorrhabda, S. sphagnicola*, *S. vinlandica*, and a new combination were observed in eight or more study sites, and except for *M. furtiva*, they were common or abundant in at least one locality. By contrast, 26 of the taxa were observed in only one or two localities. A total of five of the seven taxa previously described in the Ocala National Forest were observed there again, as well as *M. matvienkoae* var. *myakkana* in its type locality in the Myakka River.

### Taxonomic revisions and descriptions

#### *Mallomonas* section Corneae Knotek & Škaloud sect. nov.

*Type*: *Mallomonas cornea* Knotek & Škaloud sp. nov.

*Description*: The cells are elongated with bristles at each end. The external papillose layer (SEM) and interior irregular reticulum (TEM) are features shared by every type of siliceous scale. The papillae coverage is irregular, with the exception of the anterior flange, where they may be grouped in rows. There is no hood formed as the V‐rib rises smoothly. The anterior submarginal rib may have thorn‐like features that vary in prominence. The bristles are smooth, moderately curved, and have bifurcated ends.

#### *Mallomonas cornea* Knotek & Škaloud sp. nov.

**FIGURE 4:**
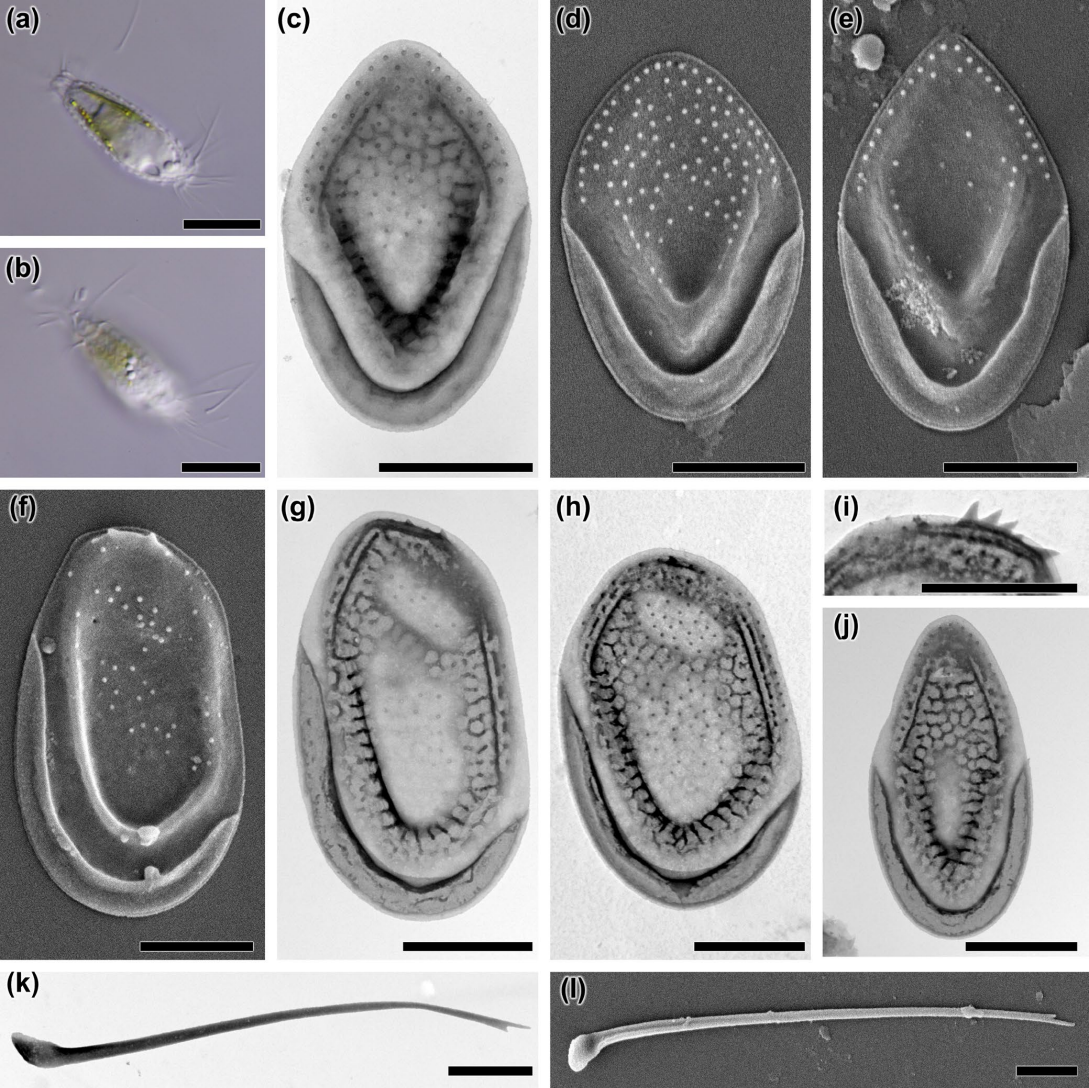
*Mallomonas cornea*, sp. nov., strain US_46H. (a, b) whole cell with scales and bristles, (c–e) body scales, (f–h) dome‐bearing scales, (i) close‐up view of thorn‐like structure located on the distal part of anterior submarginal rib, (j) rear scale, (k–l) bristles. Images taken by (a, b) LM, (c, g–k) TEM, (d–f, l) SEM. Scalebar (a, b) = 10 μm, (c–l) = 1 μm.

*Description*: The cells are ellipsoidal and remarkably elongated with dimensions of 22–30 × 6–8 μm. The bristles are limited to the anterior and posterior ends of the cell (Figure 4a). Scales are arranged perpendicular to the longitudinal axis of the cell (Figure 4b). There are three types of siliceous scales (body, terminal, and bristle‐bearing scales), all of them with an outer papillose layer (SEM) and internal irregular reticulum (TEM). The body scales are 2.3–2.9 μm long and 1.5–1.8 μm wide, ovoid with a variously pointed distal end, and lack a dome (Figure 4c–e). Papillae cover most of their smooth surface irregularly, except for the anterior flange, where they are often arranged in one or two rows. The V‐rib rises smoothly without forming a hood. The largest scales are located at both ends of the cell and are 2.9–3.9 μm long and 1.8–2.3 μm wide. They differ by a bluntly terminated distal end with a shallow dome, and their overall shape is rather oblong (Figure 4f–h). Varyingly prominent thorn‐like structures may be present on the distal part of the anterior submarginal rib (Figure 4i). At present, it is not known whether bristle‐bearing scales originating from the apical and posterior ends of the cell can be distinguished. Terminal scales are 2.5–3 μm long and 1.3–1.6 μm wide. They have a shallow dome, are much narrower and more elongated, and are most likely located at the very posterior end of the cell (Figure 4j). Bristles are 6–10 μm long, slightly curved, smooth, and have bifurcated ends (Figure 4k,l). Cysts were not observed.

*Diagnosis*: The internal reticulation, overall shape, and arrangement of secondary structures on the body scales may resemble species of *Mallomonas insignis*, but the morphological characteristics of the siliceous scales of *M. cornea* make this species easy to distinguish. The species with the most similar scale morphology to the bristle‐bearing scales of *M. cornea* are probably *M. acidophila* and *M. preisigii*. These species are similar in internal reticulation, irregular papillae coverage, shallow domes shifted to the side, and bifurcated bristles (Gusev et al., 2022; Gusev, Shkurina, & Huan, 2023). *Mallomonas cornea*, on the contrary, is distinguished by its well‐developed internal reticulation, the shape of the body scales, a V‐rib that does not form a hood, and longer and thinner bristles.

*Holotype*: Portion of a single gathering of cells on a coated TEM grid, deposited at the Culture Collection of Algae of the Charles University in Prague (CAUP) as the item TYPE‐US 46H

*Etymology*: The specific epithet “cornea” refers to the internal reticulum of siliceous scales, which resemble the cross section of irregular hexagonal cells of the corneal endothelium.

*Type locality*: Grasshopper Lake, Florida, United States (29°08′04.0″ N, 81°37′11.3″ W)

*Distribution*: Currently only known from Ocala National Forest (Florida, United States) and Belize (*Mallomonas*. sp. 1; Carty & Wujek, 2003)

#### *Mallomonas joergenii* Knotek & Škaloud sp. nov.

**FIGURE 5:**
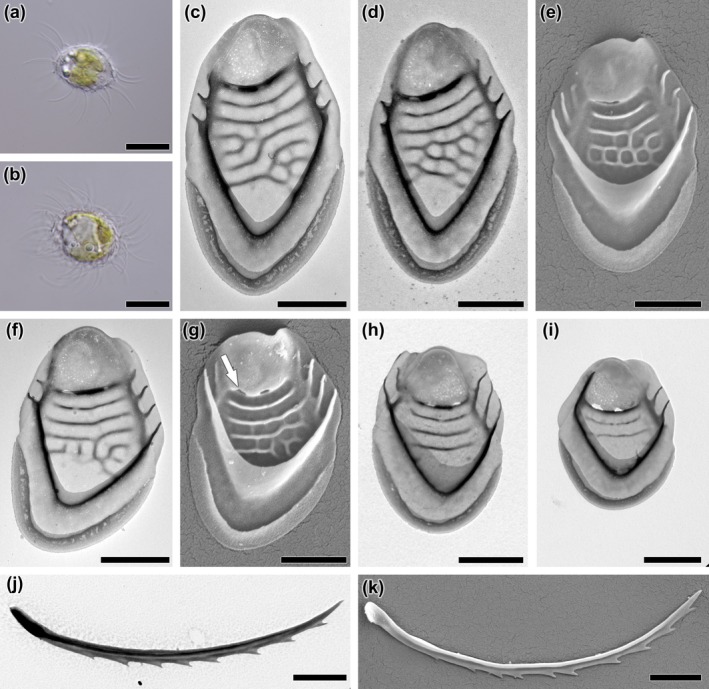
*Mallomonas joergenii*, sp. nov., strain US_58L. (a, b) whole cell with scales and bristles, (c–e) body scales, (f–g) apical scales; the white arrow points to a hole that may occur at the posterior border of the dome, (h‐i) rear scales, (j, k) bristles. Images taken by (a, b) LM, (c, d, f, h–j) TEM, (e, g, k) SEM. Scalebar (a, b) = 10 μm, (c–k) = 1 μm.

*Description*: The cells are ovoid or broadly ellipsoidal with dimensions of 13–19 × 9–12 μm. Bristles are found on the entire cell surface (Figure 5a,b). There are three types of siliceous scales (body, apical and rear scales). All three types of scales are tripartite and oval‐shaped. The body scales are 6.9–8 μm long and 4.1–4.7 μm wide (Figure 5c–e). Apical and rear ones are more asymmetrical and smaller, 2.6–3.6 μm long and 1.9–2.5 μm wide (Figure 5f–i). The dome is prominent with a patch of minute pores, non‐patterned by any secondary structure, and sharply defined against the rest of the basal plate. Some scales may have one or two holes in the posterior border of the dome. Anterior flanges are well developed and extended forward along the sides of the dome. Up to four struts branch from the anterior submarginal ribs. The shield is devoid of pores, marked with three to seven evenly spaced, slightly curved, transverse ribs. On most scales, the ribs in the proximal part of the shield become irregular and form a varying number of ring‐like structures. The V‐rib is prominent with a distinct hood without internal reticulation. There are no pores at the base of the V‐rib. The posterior flange and proximal border contain no struts. Robust bristles are curved with teeth on the convex edge and 5.6–8.4 μm long (Figure 5j,k). Cysts were not observed.

*Diagnosis*: The morphology of the siliceous scales of *Mallomonas joergenii* is similar to that of *M. striata*. The main feature that distinguishes these species is the number of transverse ribs in the shield area, and especially their irregularity and the formation of ring‐like structures in *M. joergenii*. Scales without irregular ribs and ring‐like structures can also be easily distinguished from *M. striata* by the absence of pores at the base of the V‐rib (Neustupa & Němcová, 2007). The ring‐like structures may resemble the circular depressions on the scales of *M. kristiansenii*. However, in *M. joergenii*, these structures never fill the entire shield area and are not so strongly silicified. Other notable differences between *M. joergenii* and *M. kristiansenii* include the ornamentation of the dome, the heavy ribs on the anterior flange, and the inner struts of the V‐rib and proximal border (Wujek & De Bicudo, 1993).

*Holotype*: Portion of a single gathering of cells on a coated TEM grid, deposited at the Culture Collection of Algae of the Charles University in Prague (CAUP) as the item TYPE‐US 58L

*Etymology*: The epithet is in honor of the Danish chrysophyte specialist Jørgen Kristiansen. The species *Mallomonas kristiansenii*, which has a similar scale morphology, was also named after him.

*Type locality*: Penner Lake, Florida, United States (29°29′27.0″ N, 81°49′18.8″ W)

*Distribution*: Currently only known from Ocala National Forest (Florida, United States) and South Carolina (*M. kristiansenii*; Wujek et al., 2004)

### *Mallomonas laureana* Knotek & Škaloud sp. nov.

**FIGURE 6:**
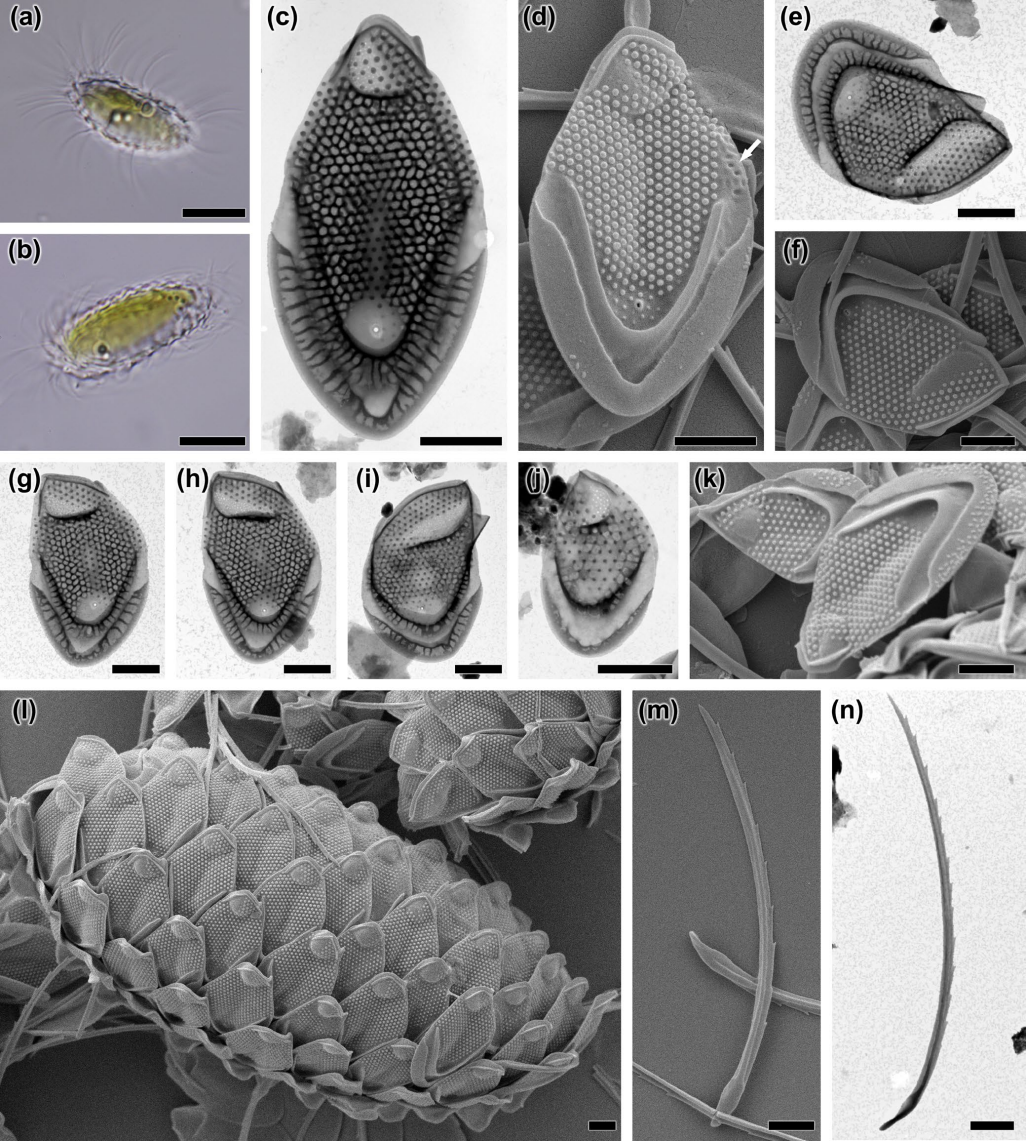
*Mallomonas laureana*, sp. nov., strain US_55K. (a, b) whole cell with scales and bristles, (c, d) body scales; the white arrow points to a small depressions at the edge of the scale, (e–i) apical scales, (j) rear scale, (k) body scale with rear scale in the background, (l) detailed view of the complete scale‐case without bristles, (m, n) bristles. Images taken by (a, b) LM, (c, e, g–j, n) TEM, (d, f, k, l, m) SEM. Scalebar (a, b) = 10 μm, (c–n) = 1 μm.

*Description*: Cells are elongate ellipsoidal or elongate ovoid with dimensions of 20–29 × 9–12 μm. Bristles are found on the entire cell surface (Figure 6a,b). All types of siliceous scales (body, apical, and rear) are tripartite. Body scales are 4.7–5.4 μm long and 2.4–2.9 μm wide and have a sub‐oval shape with narrow domes (Figure 6c,d). Apical scales are shorter and broader 3.7–4.1 × 2.3–2.7 μm with broadly oval somewhat asymmetrical domes (Figure 6e–i). The rear scales are noticeably smaller with dimensions of 2.3–3.7 × 1.9–2.3 μm and have a distinctly pointed distal end (Figure 6j,k). The siliceous scales are arranged perpendicular to the longitudinal axis of the cell (Figure 6l). Most of the scale surface is covered with closely and equally spaced papillae that fill the shield area and become smaller or completely absent at the base of the V‐rib, where there is a single circular pore. This pore may be absent in the smallest rear scales (Figure 6j). Papillae also fill the anterior flange and the dome, where up to half of the surface may remain smooth. The distal edge of the dome is bordered along its entire length by a distinctive rib formed by two arms that join at varying acute angles, depending on the position of the scale on the cell. Beyond the rib is a fine lip. The scales are filled with irregular internal reticulation in an area similar to that covered by the papillae. The only exceptions are the portion of the dome area, the margins of the anterior flange, and the base of the V‐rib. The V‐rib is prominent with internal struts. The posterior flange is narrow and smooth. The proximal border is wide with internal struts. Bristles are 7–12 μm long, curved, slowly tapering to a fine point, and unilaterally serrate with short and pointed teeth (Figure 6m,n). Cysts were not observed.

*Diagnosis*: *Mallomonas laureana* most closely resembles *M. rasilis*, *M. camerunensis*, and *M. skvortsovii*, but several differences in scale morphology can be observed between these species. The scales are easily distinguished from *M. rasilis* by internal reticulation, which is clearly visible in the scales of *M. laureana* when observed by TEM (Figure 6c). However, the absence of TEM images for *M. camerunensis* prevents confirmation of whether it also possesses internal reticulation. Additional distinguishing features are on the dome. In *M. rasilis*, small lateral ribs are present on each side of the dome, whereas in *M. laureana* and *M. camerunensis*, the ribs extend around the entire distal part of the dome. Notably, *M. camerunensis* is characterized by the presence of multiple ribs on the dome (Piątek, 2015). When observing in SEM, it is necessary to pay attention to the size of the scales and to the marginal parts of the scales, where small depressions may be visible in *M. laureana* (Figure 6d). The body scales of *M. laureana* (4.7–5.4 μm × 2.4–2.9 μm) should be larger than those of *M. rasilis* (3–4.1 μm × 2–2.5 μm) and *M. camerunensis* (3.5–4.4 μm × 1.5–2.7 μm) (Dürrschmidt, 1983; Piątek, 2015). The species *M. skvortsovii* can be confused with *M. laureana* due to its internal reticulation and the papillae covering the surface of the scales. However, on closer observation, the internal reticulation is much finer and more regular in *M. skvortsovii*, and the papillae are smaller and more numerous. The internal reticulation of the V‐rib and proximal border is also more regular in *M. skvortsovii*. Another characteristic is the dome, which is larger in *M. skvortsovii*, with parallel ribs and no papillae cover. In addition, the scales of *M. skvortsovii* are noticeably larger (6–6.7 μm × 3.2–3.5 μm; Gusev et al., 2016).

*Holotype*: Portion of a single gathering of cells on a coated TEM grid, deposited at the Culture Collection of Algae of the Charles University in Prague (CAUP) as the item TYPE‐US 55 K

*Etymology*: The epithet “laureana” refers to Laura Lake in the Ocala National Forest (Florida, United States), where the species was discovered.

*Type locality*: Laura Lake, Florida, USA (29°25′02.3″ N, 81°40′54.2″ W)

*Distribution*: Currently only known from Ocala National Forest (Florida, United States)

### *Mallomonas poseidonii* (Siver) Knotek & Škaloud comb. et stat. nov.

*Basionym*: *Mallomonas corymbosa* Asmund var. *poseidonii* Siver (1999), Nordic Journal of Botany, *19*(1): 122–123.

**FIGURE 7:**
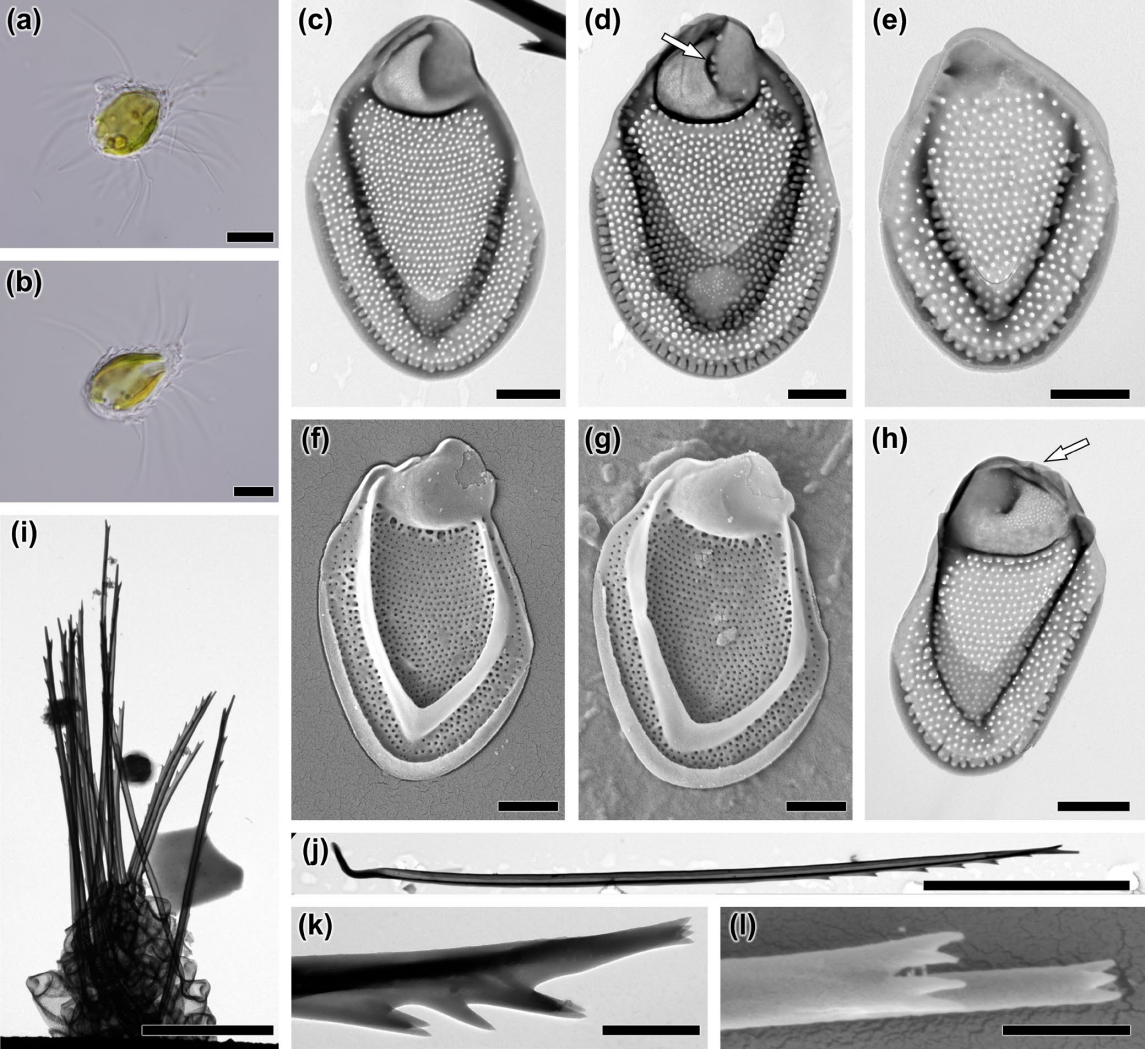
*Mallomonas poseidonii*, comb. et stat. nov., strain US_53J. (a, b) whole cell with scales and bristles, (c–g) body scales, (d) body scale; the white arrow points to dome wits several circular pits, (e) dome‐less body scale, (h) rear scale; the white arrow points to spines on dome, (i) bristles forming a corymb‐like structure on dry preparation, (j) whole body bristle, (k, l) close‐up of multifurcated teeth on bristle serration. Images taken by (a, b) LM, (c–e, h–k) TEM, (f, g, l) SEM. Scalebar (a, b, i, j) = 10 μm, (c–h) = 1 μm, (k, l) = 0.5 μm.

*Observations*: Cells are large, elongate to ovoid with dimensions 17–50 × 9–25 μm. Bristles do not cover the posterior portion of the cell (Figure 7a,b). Siliceous scales are large 4.5–7.8 μm long and 3–7 μm wide (Figure 7c–h). The dome is vertically divided into two halves. One is thinner and contains fine pores. The other is thicker and may contain several circular pits (Figure 7d). Several scales are dome‐less (Figure 7e). The smaller asymmetrical rear scales may have small rudimental spines on the dome (Figure 7h). The scales have a secondary layer with slightly larger pores than those on the basal plate. The exception is the area at the base of the V‐rib, where the secondary layer is absent. The V‐rib is prominent with a few internal struts. The posterior flange is smooth. The proximal border is narrow with internal struts. In dry preparations, the bristles cluster in a typical corymb‐like structure (Figure 7i). The apical bristles are short, curved, and serrated; the body bristles are straighter with serration only on the distal part (Figure 7j). The length of the bristles ranges 12–55 μm. Torsion of the bristle causes the distal tooth to point in the opposite direction of the remaining serration. In some of the bristles, the teeth in the serration are multifurcated, but usually split into three segments (Figure 7k,l). Cysts were not observed.

*Diagnosis*: Differs from *Mallomonas corymbosa* only in the presence of multifurcated bristle teeth

*Epitype*: Portion of a single gathering of cells on a coated TEM grid, deposited at the Culture Collection of Algae of the Charles University in Prague (CAUP) as the item TYPE‐US 53 J

*Distribution*: Currently only known from Ocala National Forest (Florida, United States; Siver, 1999; Siver & Wujek, 1999; Siver & Lott, 2006)

### Phylogenetic position of new *Mallomonas* taxa

**FIGURE 8:**
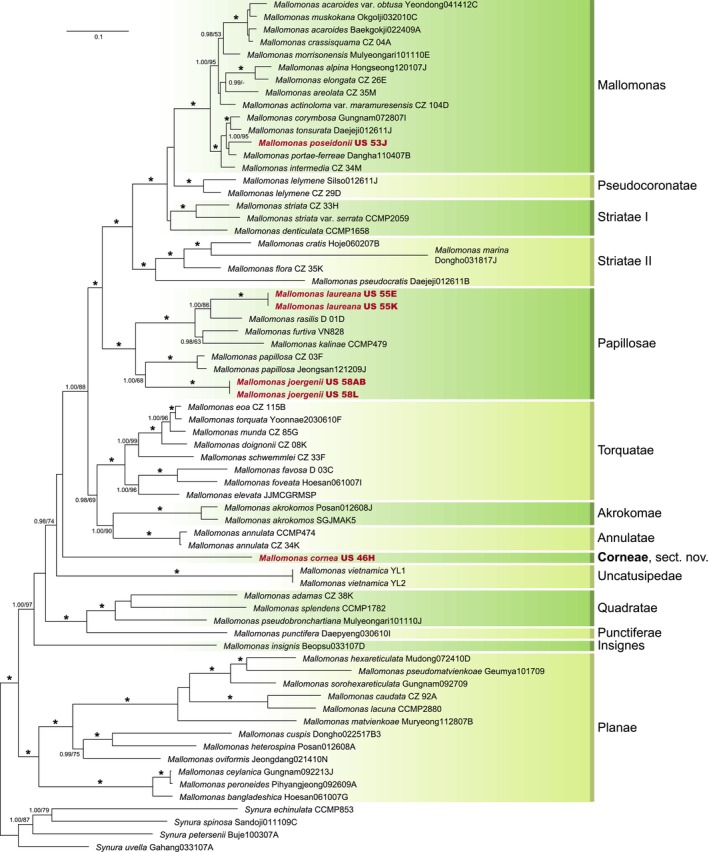
Phylogeny of the genus *Mallomonas* based on concatenated nuclear (nu) SSU rDNA, nu LSU rDNA, plastid (pt) *rbc*L, pt *psa*A, and pt LSU rDNA gene sequences. Values at the nodes indicate statistical support estimated by two methods: MrBayes posterior node probability (left) and maximum likelihood bootstrap (right). The nodes receiving the highest statistical support (1.00/100) are marked with asterisks. Scale bar represents the expected number of substitutions per site. Bold text indicates newly sequenced strains.

The phylogenetic tree of the genus *Mallomonas*, constructed on the basis of concatenated nu SSU rDNA, nu LSU rDNA, pt. *rbc*L, pt. *psa*A, and pt. LSU rDNA gene sequences (Figure 8), has a similar topology to recently published phylogenies (Gusev, Martynenko, et al., 2024; Hao et al., 2024; Martynenko et al., 2024). Most of the lineages have nine sections that were already defined based on siliceous scale and bristle morphology (Kristiansen, 2002). The exception is the paraphyletic section Striatae, which was divided into Striatae I and Striatae II monophyletic clades.

Strain US_53J of the newly described species *Mallomonas poseidonii* was closely related to *M. portae‐ferreae* (1.00/95). Together with *M. intermedia*, *M. tonsurata*, and *M. corymbosa*, they formed a strongly supported lineage within the Mallomonas section (1.00/100). *Mallomonas laureana* strains (US_55E, US_55K) and *M. joergenii* strains (US_58L, US_58AB) were nested in the Papillosae section. For *M. laureana*, its sister species was *M. rasilis* (1.00/86) and for *M. joergenii*, it was *M. papillosa* (1.00/68). Strain US_46H of *M. cornea* formed a single clade, separated from the other *Mallomonas* taxa. As this is a rather old evolutionary lineage, a new section Corneae has been described.

## DISCUSSION

### Expanding knowledge of Synurales biodiversity in Florida

The genera *Mallomonas* and *Synura* are among the most diverse in the class Chrysophyceae. *Mallomonas* contains over 250 accepted taxa and *Synura* over 50, and this number increases every year (Guiry & Guiry, 2024). Data from various studies describing the occurrence of taxa in different parts of the world have shown that there are biodiversity hotspots where Synurales species richness is significantly higher than elsewhere in the world (Gusev, Martynenko, et al., 2023; Němcová et al., 2012). These regions also often serve as type localities for newly described species, some of which have not been seen elsewhere (Barreto, 2001; Gusev et al., 2016; Gusev & Martynenko, 2022). Florida is certainly home to several of these biodiversity hotspots (Siver & Wujek, 1993; Wujek & Siver, 1997; Siver & Lott, 2006). Including the species described in this study, 90 taxa of the order Synurales have already been recorded in Florida, representing about a quarter of the known diversity.

The high number of Synurales taxa recorded in Florida is driven, in part, by the 13 studies that have documented their presence since 1983 (Table S2). A series of these studies have revealed the most species‐rich areas, as well as those with rare species. This study focused on two known Florida hotspots, Myakka River State Park and Ocala National Forest (Siver & Wujek, 1993; Siver & Lott, 2006). The Myakka River State Park contained the type locality of *Mallomonas matvienkoae* var. *myakkana* (Figure 2a) and is one of the few locations outside of Australia where *Synura australiensis* has been known to be recorded (Siver & Wujek, 1993). At all sites within the State Park, *M. matvienkoae* var. *myakkana* was the dominant species. Additionally, *M*.cf. *rasilis* (Figure 2y), which differs from *M. rasilis* by its atypical rib on the dome and larger scale size, was recorded here. For *M. stellata* (Figure 3a), this represents a second recorded observation, following the identification of *M*. cf. *peronoides/stellata* by Siver and Lott (2006). Conversely, evidence of potential species loss was observed for *M. akrokomos*. This species was observed by Wujek (1984) in lower Lake Myakka and noted as rare by Siver and Wujek (1993), but could not be detected in this study.

The Ocala National Forest represents a species‐rich hotspot for Synurales biodiversity in Florida, as 21 species have their only Florida record in this area, including the results of this study (Table S2). One of them is *Mallomonas parana* (Figure 2j), for which this is the second record since its original description in Argentina (Vigna & Kristiansen, 2005). Additionally, three new species—*M. cornea*, *M. joergenii*, and *M. laureana*—have been added to the seven taxa that have their type locality here (Siver, 1994; Siver, 1999; Siver, 2002a; Siver, 2002b; Siver & Lott, 2006). Five of these seven taxa have been observed either at or near their type localities, whereas *M. delanciana* and *M. transsylvanica* f. *curvata* were not observed. The list of re‐observed species compiled in this study, which includes *M. matvienkoae* var. *myakkana* from Myakka River State Park, suggests that the species may persist at their type localities or adjacent sites even after a considerable amount of time. This highlights the possibility of recovering species directly from their type localities and enhances confidence in their further characterization (Andersen et al., 2017; Malavasi et al., 2024; Pusztai & Škaloud, 2019). A notable example is *M. poseidonii*, which was isolated near its type locality and then elevated to species level based on molecular data. However, despite detailed morphological analyses, no distinguishing features were identified to clearly differentiate its siliceous scales from those of *M. corymbosa*. Because not all bristles in *M. poseidonii* necessarily exhibit its specific morphology, we cannot be certain of the presence of *M. corymbosa* in Florida, and identification of this species remains a challenge.

In Lakes Manatee and Lee, a total of 22 and 18 species were recorded, respectively. Four species were exclusively observed at these locations during the study. *Mallomonas ouradion* (Figure 2g) was observed solely in Lake Manatee, and *M. multisetigera* (Figure 2b), *M. pseudocoronata* (Figure 2s), and *M. pseudocratis* (Figure 2t) were restricted to Lake Lee. When compared to the species' richness at other sites, Lake Lee exceeded the average. This shows that species‐rich sites can also be located outside biodiversity hotspots. Noteworthy observations have included the presence of *M. intermedia* (Figure 1t) in Grasshopper Lake, Penner Lake, and the Myakka River. This species was thought to be endemic to Europe until the discovery of a population in a small pond in the high desert of Nevada (Siver et al., 2019). At all three lakes, *M. intermedia* was rare, raising questions about its wider distribution in North America. It remains uncertain whether the species is naturally present at multiple sites but avoids detection due to its low abundance, or whether it represents a recent introduction by an unknown vector. Finally, an undescribed species, *Mallomonas* sp. (Figure 3g), was observed in Echo Lake, Grasshopper Lake, and Cowpen Pond. The scales of this species exhibit a distinctively rounded shape, resembling those of *M. asymmetrica* (Ma & Wei, 2013). Nevertheless, the absence of a secondary layer makes them more similar to the characteristics of the Alpinae series (Kristiansen, 2002). Despite these similarities, the pore size, their uniform distribution, and the overall rounded morphology of the scales do not correspond to any currently recognized species. These findings, along with the broader results of this study, indicate that the species richness in Florida may still be under‐documented and highlight the need for further research.

### New insights into the evolution of the genus *Mallomonas*

The genes sequenced here (Table S1) allowed for more comprehensive insight into the phylogeny of the genus *Mallomonas*. For *M. laureana*, the morphology of its scales strongly suggests a close relationship with *M. rasilis*, as their scales were in some cases indistinguishable. Consistently, phylogenetic analysis positioned these species as closely related. In contrast, the phylogenetic placement of the elevated species *M. poseidonii* was somewhat unexpected, as it was determined to be sister to *M. portae‐ferreae* rather than to *M. corymbosa*, of which it was previously considered a variety. This finding suggests that within the evolutionary lineage comprising *M. corymbosa*, *M. tonsurata*, *M. poseidonii*, *M. portae‐ferreae*, and *M. intermedia*, the ancestral scale morphology likely resembled that shared by *M. corymbosa* and *M. poseidonii*.

The elongated cells of the newly described species *Mallomonas cornea*, characterized by bristles at both ends, resembled some species located in the section Quadratae. At first glance, species from other sections, such as Insignes and Retrorsae, may appear similar. However, in these sections, the terminal spines result from the elongation of scales—a feature not observed in *M. cornea*. It is worth noting that *M. insignis* also shared morphological similarities with *M. cornea* in the body scales, especially the elevated area in the distal part of the scale and the overall scale shape, which were most noticeable under SEM. However, this resemblance was considerably less apparent in the case of bristle‐bearing scales. Although their position closely resembled the collar scales characteristic of section Torquatae, their morphology was most similar to that of *M. preisigii* and *M. acidophila*. *Mallomonas preisigii* was initially described as a fossil species from the Eocene lake by Siver and Lott (2012) and was later discovered as a living species in Papua New Guinea by Gusev et al. (2022). Based on the tripartite structure of the scales, shallow dome depth, distinct absence of base plate pores, and short posterior rim, its relationship to *M. calceolus* in the section Papillosae has been discussed (Siver & Lott, 2012). The possibility of belonging to the section Ouradiotae or Multisetigerae was not included in the debate. Following this discussion, the later described *M. acidophila* was also assigned to this section (Gusev, Shkurina, & Huan, 2023). However, despite morphological similarities, *M. cornea* is not placed in Quadratae, Insignes, Torquatae, or Papillosae sections based on phylogenetic analysis. Instead, it forms a deep, separate evolutionary lineage, leading to the new section Corneae. No support for the placement of this lineage prevents us from determining the relationship of this lineage to other *Mallomonas* sections. Currently, *M. cornea* is the only member of this new section, but *M. preisigii* and *M. acidophila* may be potential candidates for reclassification into this section.

The remaining newly described species, *Mallomonas joergenii*, had a scale morphology characterized by transverse ribs in the shield area, suggesting its classification within the section Striatae. However, this morphological interpretation is misleading. Phylogenetic analysis revealed that *M. joergenii* was more closely related to *M. papillosa*. Although both species shared a similar structure of struts on the anterior flange, V‐rib, posterior flange, and proximal border, *M. joergenii* was unique within this section due to the presence of shield ribs. This will further complicate, or even prevent, the identification of common morphological traits among species currently classified within this section and highlights the need for a revision of the phylogeny of the genus. It also places *M. punctostriata* of section Striatae in a challenging position, as it possesses both ribs and papillae in the shield area (Gusev & Kulikovskiy, 2020). Papillosae is therefore another section where the shield ribs evolved independently (Němcová et al., 2024). Notably, shield ribs are among the most recently evolved morphological structures, first appearing on scales about 60 million years ago (Čertnerová et al., 2019). This raises an intriguing question: Why do certain species apparently reject their original shield ornamentation and replace it with ribs or rib‐like structures? The hypothesis that rib ornamentation in the shield area enhances the overall mechanical resistance of scales appears unlikely (Knotek & Škaloud, 2023). However, molecular phylogenetic analysis offers a robust framework for investigating this and other related questions (Čertnerová et al., 2019; Jadrná et al., 2021; Němcová et al., 2024).

The findings discussed above highlight the complexity of species diversification within the genus *Mallomonas*. Although phylogenetic trees based on scale and bristle morphology often match with those based on gene sequences, there are instances where unexpected evolutionary events are revealed, including those discussed in this study. These discrepancies underscore the importance of using sequence data to gain a more comprehensive understanding of the genus. Obtaining sequence data for genetically yet uncharacterized taxa is crucial. Integration of sequence data with other observations not only helps clarify the internal classification of the genus, but also provides deeper insights into its evolutionary history. Such an approach will enhance our understanding of the genus diversification, adaptation mechanisms, and historical biogeography, ultimately contributing to a more nuanced and complete evolutionary framework.

### Effect of climate on scale ornamentation

The siliceous scales and bristles of silica‐scaled chrysophytes exhibit diverse shapes and forms. Despite this morphological variability, their potential adaptive functions remain poorly understood. However, evidence from studies and observations indicates that abiotic factors may significantly influence their morphology. For instance, pH has been shown to impact not only the general shape and silicification of scales but also to restrict the distribution of morphologically similar and closely related species (Gavrilova et al., 2005; Němcová & Pichrtová, 2012; Siver, 1989). Temperature is another critical factor, primarily influencing the overall size of scales (Němcová et al., 2010). Additionally, Siver and Skogstad (1988) reported that temperature can drive changes in the complex morphology of siliceous bristles. They observed that the bristles of *Mallomonas crassisquama* exhibited a serrated form at temperatures below 12°C, while adopting a helmeted shape at temperatures above 15°C. However, the underlying mechanisms behind this temperature‐dependent morphological adaptation were not further explored.

Temperature also appears to be a factor in the distribution of species characterized by internally reticulated scales. In temperate regions, examples of such species include *Mallomonas adamas*, *M. allorgeii*, and *M. lychenensis* from the section Quadratae; *M. matvienkoae*, *M*. *okhapkinii*, and *M. pseudomatvienkoae* from section Planae; *M. insignis* from section Insignes; *M. annulata* from section Annulatae; and *M. favosa*, *M*. foveata, and *M. gemina* from section Torquatae. In contrast, tropical and subtropical regions host a much greater diversity of these species. Examples include *M. fenestrata*, *M. fimbriata*, *M. gusakovii*, *M. parana*, *M. pseudobronchartiana*, *M. splendens*, and *M. velari* from section Quadratae; *M. bronchartiana*, *M*. hexareticulata, *M*. lamii, M. limbata, *M. loricata*, *M. matvienkoae*, *M. okhapkinii*, *M*. paragrandis, M. *sorohexareticulata*, and *M. stellata* from section Planae; *M. favosa*, *M. foveata*, *M. fragariformis*, *M. gemina*, *M. hippocrepica, M. kornevae*, and *M. lusca* from section Torquatae; *M*. minuscula from section Pumiliae; and *M*. laureana, *M. skvortsovii*, and *M. spinosa* from section Papillosae. This distribution pattern indicates that species with internally reticulated scales are notably more common in tropical or subtropical climates. For example, all species in the section Papillosae with pronounced internal reticulation (*M. laureana*, *M. skvortsovii*, and *M. spinosa*) live exclusively in warm water tropical areas (Gusev, 2012; Gusev et al., 2016; Gusev, Martynenko, et al., 2023). One possible explanation for this species distribution is related to UV irradiance. A study by Němcová et al. (2024) revealed that species with thick‐walled, internally reticulated scales, such as *M. adamas* and *M. splendens*, exhibited significantly greater resistance to harmful UVB radiation. This aligns with the observation that tropical regions, where UV intensities are highest, are also rich in species with internally reticulated scales (Blumthaler et al., 1997). However, further research is needed to clarify this relationship, the associated mechanisms, and the wider ecological implications.

## AUTHOR CONTRIBUTIONS

**Petr Knotek:** Conceptualization (equal); data curation (equal); formal analysis (equal); funding acquisition (lead); investigation (lead); methodology (supporting); writing – original draft (lead); writing – review and editing (lead). **Martin Pusztai:** Resources (equal); writing – review and editing (supporting). **Iva Jadrná:** Data curation (equal); writing – review and editing (supporting). **Pavel Škaloud:** Conceptualization (equal); data curation (equal); formal analysis (equal); methodology (lead); resources (equal); supervision (lead); writing – original draft (supporting); writing – review and editing (supporting).

## Supporting information


**Table S1.** GenBank accession numbers of genes used in phylogenetic analysis.

**Table S2.** A complete list of observed species and their abundance at each study site, including their history of occurrence in Florida.
